# Supplementary material for: MaPom1, a Dual-Specificity Tyrosine Phosphorylation-Regulated Kinase, Positively Regulates Thermal and UV-B Tolerance in Metarhizium acridum
Source: Int J Mol Sci. 2024 Nov 5;25(22):11860. doi: 10.3390/ijms252211860 (PMC11594272; doi:10.3390/ijms252211860)
Supplement: Supplementary file 1 [file ijms-25-11860-s001.zip › ijms-3246971-supplementary.pdf]

MaPom1, a Dual-Specificity Tyrosine Phosphorylation-Regulated Kinase, Positively Regulates Thermal and UV-B Tolerance in *Metarhizium acridum*

Yu Zhang [1,2,3,4,†], Lei Song [1,2,3,4,†] and Yuxian Xia [1,2,3,4,*]

[1] School of Life Sciences, Chongqing University, Chongqing 401331, China; 18838933368@163.com (Y.Z.); songlei19960404@163.com (L.S.)

[2] Chongqing Engineering Research Center for Fungal Insecticides, Chongqing 401331, China

[3] Key Laboratory of Gene Function and Regulation Technologies Under Chongqing Municipal Education Commission, Chongqing 401331, China

[4] National Engineering Research Center of Microbial Pesticides, Chongqing 401331, China

* Correspondence: yuxianxia@cqu.edu.cn; Tel.: +86-185-2300-1964

† These authors contributed equally to this work.

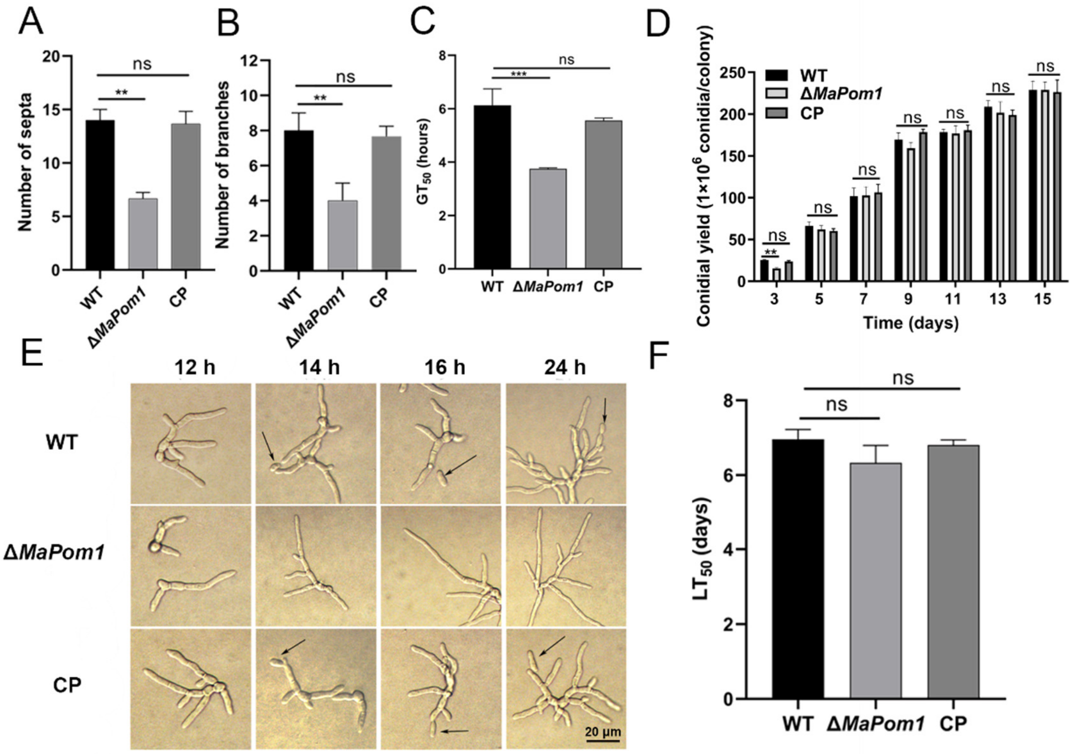

Figure S1 Analysis of germination, growth, conidial yield, and virulence. (**A**) Number of speta in WT, Δ*MaPom1*, and CP on 1/4 SDAY medium. (**B**) Number of branches in WT, Δ*MaPom1*, and CP on 1/4 SDAY medium. (**C**) Conidial yields of WT, Δ*MaPom1*, and CPon 1/4 SDAY medium. (**D**) Conidiation of WT, Δ*MaPom1*, and CP on 1/4 SDAY medium. Black arrows represent the fallen conidia. Error bars represent standard deviations based on three independent replicates. Asterisks indicate significant differences at p <0.01 (**), while "ns" indicates no significant differences, and different lowercase letters indicate significant differences by one-way ANOVA and Tukey test.

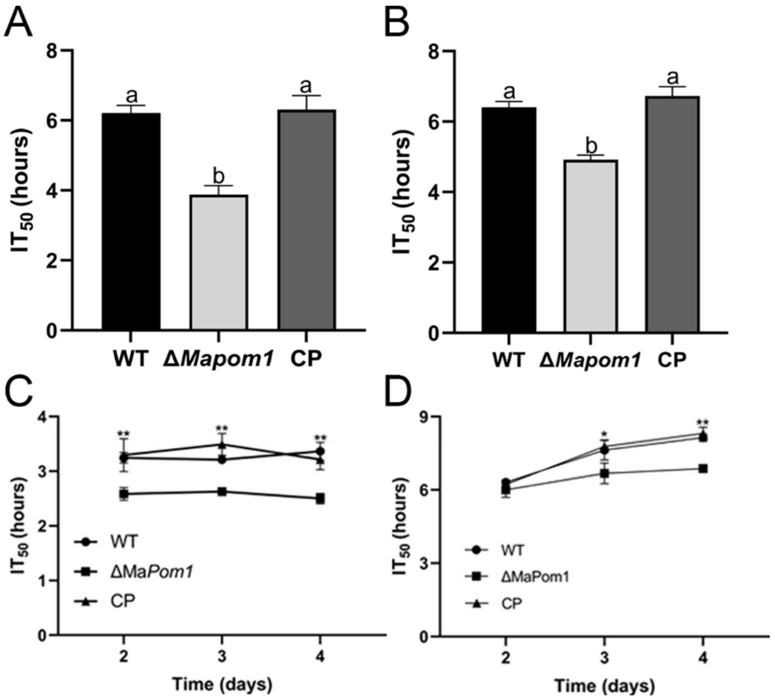

Figure S2 Half-inhibition time (IT_{50}) under heat shock and UV-B treatments. (**A**) IT_{50} of heat shock treatment. (**B**) IT_{50} of UV-B irradiation. (**C**) IT_{50} of UV-B irradiation of conidia at different stages. (**D**) IT_{50} of heat shock treatment of conidia at different stages. Error bars represent standard deviations based on three independent replicates. Asterisks indicate significant differences at P <0.05 (*), P <0.01 (**), and different lowercase letters indicate significant differences by one-way ANOVA and Tukey test.

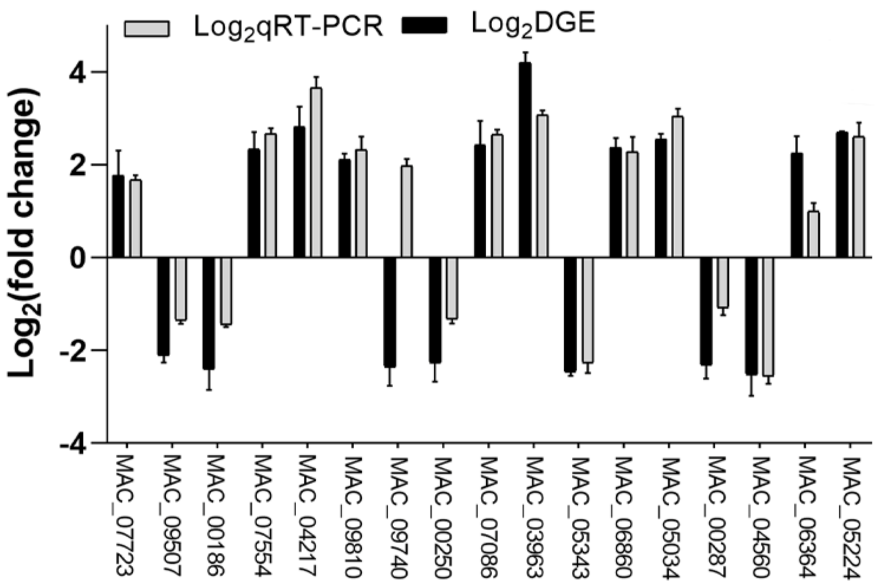

Figure S3 Quantitative verification of DEGs in digital expression spectrum

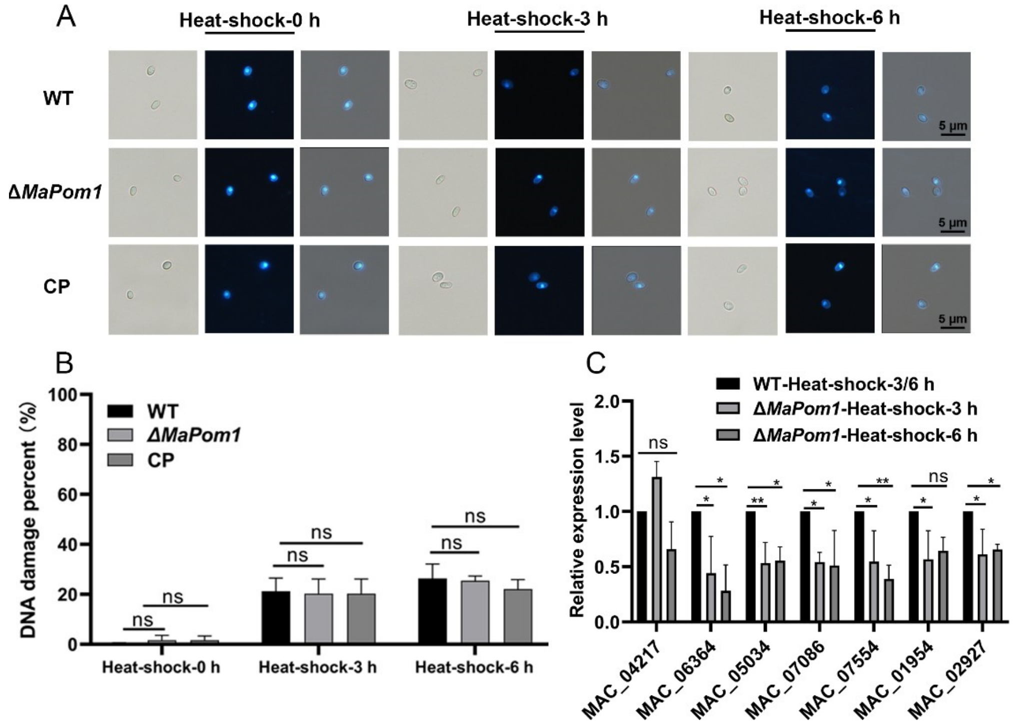

Figure S4 Analysis of heat shock tolerance regulatory mechanisms. (**A**) DNA staining with DAPI after heat shock treatment for various durations. “Heat-shock-0 h”

indicates no heat-shock treatment, "heat-shock-3 h" indicates heat-shock treatment for 3 h, and "heat-shock-6 h" indicates heat-shock treatment for 6 h. Conidia were stained immediately with DAPI after treatment. (**B**) Percentage of DNA damage after heat shock treatment for different durations. (**C**) Relative expression levels of HSP genes after 3 h and 6 h of heat shock treatment. "WT-heat shock-3/6 h" indicates conidia from WT treated for 3 or 6 h, respectively. "Δ*MaPom1*-heat shock-3 h" and "Δ*MaPom1*-heat shock-6 h" indicate conidia of Δ*MaPom1* treated for 3 h or 6 h, respectively. The relative expression levels of different genes in the WT were normalized to 1 after different treatments. All the images were acquired at a fixed exposure time. Error bars represent standard deviations based on three independent replicates. Asterisks indicate significant differences at P <0.05 (*), P <0.01 (**), while "ns" indicates no significant differences by one-way ANOVA and Tukey test.

Table S1 Primers used in this study

Primers	Sequence (5′-3′)	Remark
MaPom1-LF	GACCGCAACTGCCTGGAT	Construct *MaPom1* knock out vector
MaPom1-LR	CGGGAGGGAGGGTAGATT	
MaPom1-RF	ATCCAACGCTGGCACAAG	
MaPom1-RR	CGATTCCCACAGCCCTAC	
MaPom1-VF	GTCCCAATGCGACGTGCAGA	Screen *MaPom1* knock out strains
PT-R	CAGCCAAGCCCAAAAAGTG	
MaPom1-VR	CTCACACCTCGTTGCGCCCG	
Bar-F	GCTCTACACCCACCTGCT	
MaPom1-TF	TAACAGTTGACCTCCAAAATCG	Southern blot probe
MaPom1-TR	TTTTAGATGAGTGGCACTTTCC	
MaPom1-CP-LF	GACGGCCAGTGCCAAGCTCGCCCAAAACGTACCAAATGT	Construct *MaPom1* complemented vector
MaPom1-CP-LR	CCTTGCTCACCATGGATCCCT	

	CGTCGGCTCATTGCGG	
MaPom1-CP-VF	GGCATGCCCATCGATATGTG	Screen *MaPom1* complemented strains
EGFP-VR	CGATGCGGTTCACCAGGGTGT	
GAPDH-QF	GACTGCCCGCATTGAGAAG	
GAPDH-QR	AGATGGAGGAGTTGGTGTTG	
MaPom1-qF	ACCATGGCCCAATGCCATAA	Quantitative PCR primers
MaPom1-qR	TCCAGGAGTTCCGTCGAGAT	

Table S2 Results of DEGs in conidia resistance

Gene ID	Log_2Ratio (Δ*pom1*-3 days vs. WT-3 days)	Up or down regulation	Product name
MAC_03963	4.399100987	Up	Multidrug resistant protein
MAC_02027	4.187734451	Up	Cytochrome P450
MAC_08098	4.023089911	Up	Endoglucanase
MAC_06641	3.698732564	Up	Allergen
MAC_03476	3.317338368	Up	Coatamer subunit protein
MAC_04217	3.253287244	Up	Heat shock protein 30
MAC_05811	3.206752006	Up	Hypothetical protein
MAC_08501	3.120841777	Up	Hypothetical protein
MAC_06007	2.875893748	Up	Apoptosis-inducing factor, putative
MAC_04876	2.804486573	Up	Hypothetical protein
MAC_06364	2.712362678	Up	Heat shock protein 30
MAC_05034	2.666710627	Up	Heat shock protein 101
MAC_05449	2.466205678	Up	Epoxide hydrolase 1
MAC_02303	2.45961124	Up	Protein bli-3
MAC_07086	2.346661514	Up	Heat shock protein 78 precursor
MAC_04752	2.283385295	Up	Hypothetical protein
MAC_06955	2.277201232	Up	Hypothetical protein
MAC_08010	2.239412578	Up	Hypothetical protein
MAC_09810	2.227092276	Up	Membrane transporter
MAC_06833	2.20533441	Up	Trypsin-related protease
MAC_09491	2.189577665	Up	Hypothetical protein
MAC_09520	2.16917843	Up	Amino acid transporter, putative
MAC_07723	2.168402328	Up	DUF1264 domain protein
MAC_09143	2.165645481	Up	Putative trehalose-6-phosphate synthase/trehalose phosphatase

Gene ID	Log_2Ratio (Δ*pom1*-3 days vs. WT-3 days)	Up or down regulation	Product name
MAC_06365	2.159179902	Up	Fungal specific transcription factor, putative
MAC_04815	2.157669362	Up	Hypothetical protein
MAC_07554	2.133973612	Up	Heat shock protein 30
MAC_09408	2.113073618	Up	NADP-dependent alcohol dehydrogenase C
MAC_05167	2.112821041	Up	Cytochrome c biogenesis factor（heat shock protein STI1）
MAC_04840	2.08958725	Up	Hypothetical protein
MAC_04197	2.072884602	Up	Nucleotide exchange factor SIL1
MAC_00595	2.068179019	Up	Cytochrome P450, putative
MAC_02740	2.038635947	Up	3-methyl-2-oxobutanoate hydroxymethyltransferase
MAC_05224	2.036325536	Up	Hsp70 nucleotide exchange factor (Fes1)
MAC_09521	2.028735033	Up	Pyridoxal-dependent decarboxylase domain protein
MAC_05128	2.026316956	Up	Acyl-coenzyme A oxidase
MAC_03233	2.025688816	Up	DUF1264 domain protein
MAC_05133	2.022714604	Up	Cell wall protein
MAC_06615	2.018273814	Up	Putative cyclic nucleotide gated channel beta 1
MAC_07720	2.016871481	Up	Mannose-6-phosphate isomerase
MAC_09310	2.014241929	Up	Conidial pigment polyketide synthase PksP/Alb1

Gene ID	Log$_2$Ratio (Δ*pom1*-3 days vs. WT-3 days)	Up or down regulation	Product name
MAC_00200	-4.364938676	Down	Pfs, NACHT and WD domain protein
MAC_09009	-4.359970651	Down	Hypothetical protein
MAC_02688	-4.181809662	Down	Hypothetical protein
MAC_01948	-3.660854403	Down	Hypothetical protein
MAC_00247	-3.44094969	Down	Hypothetical protein
MAC_00287	-2.94835378	Down	Penicillin-binding protein, putative
MAC_06626	-2.696152614	Down	Hypothetical protein
MAC_02889	-2.637923581	Down	Hypothetical protein
MAC_06676	-2.633438163	Down	Hypothetical protein
MAC_00183	-2.56307876	Down	Alcohol dehydrogenase, putative
MAC_05783	-2.558784652	Down	Hypothetical protein
MAC_05343	-2.555934334	Down	RNA-binding protein, putative
MAC_02283	-2.531097935	Down	NAD dependent epimerase/dehydratase, putative
MAC_02230	-2.454903081	Down	Hypothetical protein
MAC_09163	-2.435778234	Down	Hypothetical protein
MAC_09740	-2.428712492	Down	Monocarboxylate permease, putative
MAC_02284	-2.360447125	Down	Hypothetical protein
MAC_03210	-2.345339314	Down	Thiamine pyrophosphokinase
MAC_00248	-2.293320074	Down	Hypothetical protein
MAC_06860	-2.286418622	Down	Nonribosomal peptide synthase, putative
MAC_00179	-2.266813385	Down	*O*-methyltransferase, putative

Gene ID	Log_2Ratio (Δ*pom1*-3 days vs. WT-3 days)	Up or down regulation	Product name
MAC_09507	-2.261181162	Down	Hydrophobin
MAC_00181	-2.258049954	Down	Hypothetical protein
MAC_05044	-2.248849178	Down	Hypothetical protein
MAC_01245	-2.23104724	Down	Hypothetical protein
MAC_06623	-2.230634931	Down	Cytochrome P450 phenylacetate 2-hydroxylase, putative
MAC_07851	-2.20376267	Down	Hypothetical protein
MAC_00250	-2.178019372	Down	LEA domain containing protein
MAC_09733	-2.172151951	Down	Hypothetical protein
MAC_04560	-2.157836454	Down	Efflux pump antibiotic resistance protein, putative
MAC_00186	-2.125847252	Down	C6 transcription factor, putative
MAC_05836	-2.109353807	Down	Hypothetical protein
MAC_05216	-2.102568616	Down	Hypothetical protein
MAC_00459	-2.094212242	Down	Hypothetical protein
MAC_02291	-2.087785098	Down	Hypothetical protein
MAC_00237	-2.080294676	Down	MHYT domain signaling protein, putative
MAC_09055	-2.06961446	Down	Hypothetical protein
MAC_05040	-2.065657445	Down	Candidapepsin-4 precursor
MAC_08672	-2.064535964	Down	Hypothetical protein
MAC_05185	-2.054627562	Down	Putative asparaginase like 1
MAC_04576	-2.042377813	Down	HMG box protein, putative
MAC_05043	-2.042358641	Down	Hypothetical protein
MAC_00180	-2.005781231	Down	FAD dependent oxidoreductase family protein

Table S3 Primers used in qRT-PCR analysis of DEGs

Gene ID	Gene product description	Primer sequence (5′-3′)

MAC_04217	Heat shock protein 30	ACCCCAACTACCACCACAGC<br>TGGGGAAGTTGAAGCTGCGA
MAC_06364	Heat shock protein 30	CGGCATGAACAAGGACACGG<br>CGGGCTTCTCGGTCACATCT
MAC_05034	Heat shock protein 101	CTGCTCTCAATGCTGCTGGC<br>GATACCGGTCCAACGAGCCA
MAC_07086	Heat shock protein 78 precursor	GCAGCGTCTGGATGACAAGC<br>CCCGTTGCCAATCTCGTTCG
MAC_07554	Heat shock protein 30	TCCCAACACAAGGCGACTGT<br>CCGATGCTGCGCTCAGAAAG
MAC_05224	Hsp70 nucleotide exchange factor (Fes1)	AATTACCAGCCCGCCATGGA<br>GAATAACAGCGTCCACGGCG
MAC_06860	Nonribosomal peptide synthase, putative	CTATCACGGCGTGGGATGGT<br>CATCACGATTGGCAGCACCC
MAC_00250	LEA domain containing protein	GTTGGGCGGTTTGGGACTTG<br>TTCCCGGTCAAAGCACTCGT
MAC_00186	C6 transcription factor, putative	CGTCGTCGCAGTCATCGTCATC<br>TGGCGGAGAGTGAGGAAGGATG
MAC_00287	Penicillin-binding protein, putative	CCCAACCACACAATCTCTGATCCG<br>GAAGCGTCATGGTGCCGTATCC
MAC_05343	RNA-binding protein, putative	GCGGCGGCGTGTCTAACATC<br>GGTGATTCTGGATGGTCGGTGTTG
MAC_09740	Monocarboxylate permease, putative	ACAGACCAGGCGGGCTTGAG<br>GCCACACCCCAGGTATTGAAGAAG
MAC_09507	Hydrophobin	CGCTGTTGCCGCTCTCCTTG<br>GGCCGATAACACCGAGAACAAGAG
MAC_04560	Efflux pump antibiotic resistance protein, putative	CACGACCAAGAGCCAACTCAACC<br>CGGAACCATGCCAGGAACCATATC
MAC_03963	Multidrug resistant protein	TGCTGTGCTATTCCGTTCCTGTTC<br>CATCGCCTTTCCTCGCCTTCTC
MAC_09810	Membrane transporter	CGAACCAGACAGCAGCAGTGAC<br>CCTGATACGGCTCCCTCCCATC
MAC_07723	DUF1264 domain protein	CTGAATGCCTTCCACGCCTACG<br>GGAGACACTGCCGAACATCACTG
MAC_04576	HMG box protein, putative	ACCGTTGTTCCTCCAGTCGTTTG<br>ACTACTTGCGTCTCTTGGGATTGC

MaPom1 cDNA Sequence

ATGCTGTCGGCACCCTCTACAAACTATTCTCTACTCTCATTCGACCGAAATC
GCGATAATGGCCATACCGCCGAATCACCTGGATCTTTCGATTTTCTCCCGTC
TGTCAGTTTTGATGAACTGCACACCAGTATCGAATCTGCTTCAACCGATTTC
AAACTTACCCAGTTCCCTTCACCCACCGGCGAAGGAAGCATTCTTGAGGG
GAAAGGTTTAATGGAAAGGAAACTACCTGAAAGGCCAATCATGACGCAGA
ACACTGGCACAACACGTGGAAGCATACCGCCTCCGCCGTCTCGACCCGGT
CGTTCAGGGTCAATCTTGCGTCGACCGAGCACCTCGAGCAGGCAGACGAG
TGTGTCCTCAGTCGTGTCTTCTTCCTCTGGTGGCACACTGGATGCCCCTTCT
GCGCCCGCAGCTATGCGAAATCGTCGCCAGAGTCAATTTCCCCCAGTTTCA
AATGCCGCCGCCCCCAGGCCACCTCGGAAATCCATGGGGCCTGGAGTCATC
GCAGATTCGGACCTGGCCGCCCGAAATGCACCTGCCAGGCGCACCAGTCT
GTTGGGAGACAAAAATGCCCCCCGGGACTCTACAAGACGGTCTATTGACG
GTGGTTCTGTAACTGGTTCAGACTCAACACGCAATTTGCCAACATCGCGGG
CAATCAAGGCAAAATCAGTTCAGCCACCTCCGCGGACGAGTCAGACGAAC
TTGCTCGGGGGGTCTACTCTTACTCCAGAGCAACACCGTTTATCGACCCTG
GCTCCTAGATCTCCACGTGTTGGCGCAAAGGCAGGTACCCCATCATCTGGC
TCTAGCAAAAGGATGTCGATGATGCCGGGCAGCCACGCCACGGGATTAGG
AGCTCGTACAATCAGCCCAACTGACACGAGAAGAATGAAACGCTTGTCCA
TGATGCCACAATCACAAAGCTTAAACATGCTTGCGAATGTGCCTCCACCTC
CTCCGCCAGTATCAATGGATGTGCGAGCGGAGTCTCGATCTCCATCCATGAT
CCCACGCAAGGCCTCCCTGACTCCTTCTTCCGCCAGAACGACCCCAGATAT
AAACAATCGAAAGTCGTACAGCTCCGGTCTCTCTGTAGGCTCTACGGCTAG
TTATAATACTGTGAGGACGTCTACGGGATCTGTTCAACCTCGTCTCCCTCTA
CCATCGTCTGCAACTCGATTGCCGGCGCCCAAGCACGGCAGCACGCACAA
CAATTTGCAAACAGACGATGATGAAGACGTTCCCCCTGTTCCTGCCATACC
CAAAGCCTATGAATCGCCAAAGGAATCCCATGCTGAGACGTACTTTATGGA
AAAGAAGAAATCGAGCCTCAACAATCTCGACTCAACGAGTATTCATAGCAA
TTCGACTAGTAGCATATCAATGCCAGTCCACCTCGAGCCTACCAAAGTTCA

GCAAAAGGCGAATGTCAGAAAGAACACCTATGCGGGGATAACCGCTGTTG
AAGAAGAGAATCAAGAAATTCAGTCTAGGAAACAGCTGGAACCACTGAGT
CTACCGCCTCTTAATATTGGGCCATTGAACTTTCCTTCCACCTCCAAAGTCA
ATGGTCAAACTTCTTCTCATCGAGATCTCAGCCCACCACCTTCACGACAAG
TTCCCAAGACTCCTACAACACCTATAACGGCATCGAAGAGTTCATTCTTCTC
CAAGAGTCGGTATGACGAAACGTTGGAACTTCCCTCTCTTAGGAGCAGTAC
CTCCGCTCATCATATCCGCCGAGTTACGCAGACCCCCCCTGATGGCATCTCT
TCAGATTCGTCCCCTAGCTTGATTGAGCCGATCCACAAGTCGAGCATCTCCC
CCTTTCTATCGTCGTCTTTGCCTAAAGGAGGCTTTGAGGCTGGGCACCTGG
AGAGGTCCAAGACTGGAGGGGATTGTGCCACCATTACAGGAGCTTTTTTGG
AGCCCAGTGCCGAACGAAAGCCTTCTGGCCCTCGAGAACTGAACAAGGCT
AAGCCTATCCCGCAGTCTCCACCAGATACTGCAGAGACTCAAGGACCTCAA
AGTCCCTCCTCCAAGACATCCCTTCGGAGAAAGCTTAGCTTGTCGTGGAAG
CGAAGCAACTCCAAGTCCGGTTCTGTTGACACGGCTGATAAATCTGGCACG
CAGCAGCCGCCAAAATCAGATGGCATGCCGCCTCCCCGGATTCCTGTCTCA
TCATCTGCTGCGGTGGGCGGTTACTCCGGTCTCAAACAGCCCAATCCTAGT
CCCATCGTAAACACGAATTCTCATGGAGCTAATGCTGATATCAGAAGACGG
AAGAGCTCGGCAGCAAGTTTAACGAATTACGGGGCCCCCAGTCGCATCAA
GAGCGATACATGGCACGCTCATCAAGAAGGACTTGACCCCATTGCTGTGCC
TTCGACCAGAAGCGCTAGTGGCGTATCGAATAAGATGGTCAAGCCCAAGTC
ATCCGTTAATGCACTCCGGACTGTTGGTTCTTGGACTACGGACCCCGATAA
AGACGATATTGCTGCTGAAGAAGAAATGCGCAAGCTAGGATCTCGACGAA
AGGAAACCGAGATTGCTGGCAGGGCATTGGACGCCTTGAAGAAGAGGGC
AACGCCAAAGGAGCGTGTGGGGTCTCAGGATGCCATACGGATTGCTATGCT
CAATATATATGAACGAGGCGAAATTATTGACTACAACGACGTATACTTTTGT
GGAACCCAAAATGCCCGGAAAGTTGTGGGAAACTTGCAGTCAGACACTCC
AAATTTTGGCTATGATGACGAACGCGGTGATTACACTATTATCCCAGGTGAT
CACCTTGCGTACAGATATGAGATTGTGGATATTCTTGGAAAGGGAAGCTTTG
GACAGGTTGTTCGGTGTATTGACCACAAACTTGGGGTTCTTGTTGCCATCA
AAATCATTAGGAACAAGAAAAGGTTCCATCAGCAAGCTCTTGTCGAGGTC

AATATTCTTCAAAAGCTTCGCGAATGGGATCCCAAGAATAAACACAGCATG
GTCAATTTCACGCAGAGTTTCTATTTCCGCGGCCACCTTTGTATCTCGACGG
AACTCCTGGATATGAACCTTTACGAGTTTATTAAGGCACATTCTTTTCGTGG
GTTCTCTTTGAGAATTATTAGGCGATTCACGAAGCAGATCCTGAGCTCTCTA
GTTCTGCTTAAGCAGCGTAAGGTCATTCATTGCGACTTGAAACCCGAAAAT
ATTCTTCTGAAGCATCCTTTGCACTCGGAAATCAAGGTGATCGACTTTGGAT
CAAGTTGTTTCGAGCATGAGAAGGTATACACGTATATCCAGTCAAGATTTTA
TCGATCCCCAGAAGTCATTCTAGGTATGACATATGGCATGCCCATCGATATGT
GGAGTGTAGGTTGTATTCTGGCCGAACTTTACACAGGGGTTCCCATTTTCCC
TGGTGAGAATGAGCAAGAGCAGCTGGCATGTATTATGGAGGTGTTTGGACC
CCCCGAGAAGCATTTGATCGAGAAGAGCACGAGAAAGAAACTATTCTTCG
ACTCTATGGGGAAACCGCGGTTGACTGTATCGTCGAAAGGCCGACGTCGTC
GTCCGTCGTCAAAGACCCTGCAGCAGGTGTTGAAATGCGACGACGAAGCT
TTCTTGGACTTCATTGCTCGTTGCCTGCGATGGGATCCTGAGAGGCGACTG
AGACCCGAGGACGCCGTTCGGCATGAATTCATCACGGGTCAGAAAGTACC
AGTCATCATCCCAAGGCCGCCAGCCAGGGAATCGTCGCCGTCGAAACGAG
TCAATCCCCTTTCGGCCCCTCGCCCCCTACCCGACCCTCCAGCTTCTATCGT
TAAAGGAGTTGGCGGTTCCATGCGGACTGGCATTAGCCCCCACAAGCCGGT
ATCTGGCACTTCAAGAAGAACCTCCGGGGCTACAGCTGCTACCGCTGCTAG
CATCAACAGACGAACCAGTGCGGGCGGTTCCATTAGCAGTATCAGCAGTCT
ACCTCGTGCCGCTGGTCGAACTGCTAGTGGCAAACAGGACCTTGCAGCAG
CTGGTGCGTCGGCCGCAATGAGCCGACGAGTATAA
